# St. Luke’s Free Hospital

**Published:** 1866-11

**Authors:** 


### ST. LUKE'S FREE HOSPITAL.

The second Annual Report of this Hospital has been received. Although this most excellent charity, by its special charter, is wholly under the control of the Protestant Episcopal Churches of Chicago, and principally supported by them, it is maintained for the needy poor of Chicago without regard to religious faith. It will be observed, that of 198 cases tabulated in the annual Report, only 33 were Episcopalians.

The Hospital is under the management of a Board of Trustees and a Board of Direction, the last being composed of ladies.

The Physician-in-Chief is John Owens, M.D. The Consulting Physician is Prof. J. Adams Allen, M. D.; Consulting Accoucheur, Prof. W. H. Byford, M. D.

There are 49 obstetrical cases recorded in the Physician's report.

Students are permitted to visit the Hospital, on applying to the attending Physician.

We can scarcely conceive of a nobler object, for which a

Christian denomination can labor outside of its special church work, than for the establishment and support of a good hospital for the benefit of the sick poor, however small it may be.

We believe it is often as great a charity to prevent mental as physical suffering. There are grades of society even among the poor, which should, to a certain extent, be respected.

There are very many ignorant poor, who, from their habits and associations through life, are perfectly content to become patients in our general hospitals, if they can be persuaded that "the doctors" will not "try experiments on them," and will employ their best skill in endeavoring to relieve them.

There are also other classes who, from their education and relations in society, without the charge of false pride, would suffer mental distress and feel degraded on being thrown into the common wards of a pauper hospital. Every practising physician is constantly observing, for instance, widows, left poor and with children, parents with large families of children, who have neither the accommodations for sickness, nor the means of paying a physician a tithe of his fees. There are respectable women about to be confined, whom circumstances have suddenly deprived of a home; there are seamstresses, shop girls and young men often disabled by sickness or accident, in large numbers in our city, who have but the doubtful comforts of a boarding-house. Such patients are often able to pay a part or even the whole of their expenses at such hospitals as we have described. The benefits which all these classes of sick poor receive at such hospitals as St. Luke's and the Hospital for Women and Children are almost incalculable.

We hope to see an increase, within certain limits, of small hospitals in each division of Chicago, under the patronage of our different churches.

The sick can then be conveyed more readily and more conveniently to themselves and friends to comfortable quarters. The medical attendants can be physicians residing near the hospitals, and easily called in cases of emergency.

We will call the attention of our readers in our next issue to the other hospitals of Chicago.

